# Plants Attract Parasitic Wasps to Defend Themselves against Insect Pests by Releasing Hexenol

**DOI:** 10.1371/journal.pone.0000852

**Published:** 2007-09-05

**Authors:** Jianing Wei, Lizhong Wang, Junwei Zhu, Sufang Zhang, Owi I. Nandi, Le Kang

**Affiliations:** 1 State Key Laboratory of Integrated Management of Pest Insects and Rodents, Institute of Zoology, Chinese Academy of Sciences, Beijing, People's Republic of China; 2 Department of Entomology, Iowa State University, Ames, Iowa, United States of America; 3 Institute for Systematic Botany, Zurich, Switzerland; Northwestern University, United States of America

## Abstract

**Background:**

Plant volatiles play an important role in defending plants against insect attacks by attracting their natural enemies. For example, green leaf volatiles (GLVs) and terpenoids emitted from herbivore-damaged plants were found to be important in the host location of parasitic wasps. However, evidence of the functional roles and mechanisms of these semio-chemicals from a system of multiple plants in prey location by the parasitoid is limited. Little is known about the potential evolutionary trends between herbivore-induced host plant volatiles and the host location of their parasitoids.

**Methodology/Principal Findings:**

The present study includes hierarchical cluster analyses of plant volatile profiles from seven families of host and non-host plants of pea leafminer, *Liriomyza huidobrensis*, and behavioral responses of a naive parasitic wasp, *Opius dissitus*, to some principal volatile compounds. Here we show that plants can effectively pull wasps, *O. dissitus*, towards them by releasing a universally induced compound, (*Z*)-3-hexenol, and potentially keep these plants safe from parasitic assaults by leafminer pests, *L. huidobrensis*. Specifically, we found that volatile profiles from healthy plants revealed a partly phylogenetic signal, while the inducible compounds of the infested-plants did not result from the fact that the induced plant volatiles dominate most of the volatile blends of the host and non-host plants of the leafminer pests. We further show that the parasitoids are capable of distinguishing the damaged host plant from the non-host plant of the leafminers.

**Conclusions/Significance:**

Our results suggest that, as the most passive scenario of plant involvement, leafminers and mechanical damages evoke similar semio-chemicals. Using ubiquitous compounds, such as hexenol, for host location by general parasitoids could be an adaptation of the most conservative evolution of tritrophic interaction. Although for this, other compounds may be used to improve the precision of the host location by the parasitoids.

## Introduction

Plants have evolved a wide spectrum of strategies to defend themselves against herbivores, including both direct and indirect defenses. Direct defenses like physical barriers or toxins derived from secondary plant metabolites prevent herbivores from feeding [1]; indirect defense of plants works by using herbivore-induced volatile compounds or extrafloral nectar to attract their natural enemies [2]–[7]. To date, nearly 2000 volatile compounds have been identified in species from over 90 plant families. These compounds are released from plant organs above or below the ground some of which induced by various biotic activities [8]. Plant volatiles play an important role in mediating the behavior of herbivores and their natural enemies [3], [5], [9]. The function of herbivore-induced volatiles as indirect plant defensive signals has received wide attention at the infochemical, biochemical, genetic, and molecular levels [1]. It is well known that herbivore-damaged plants emit some green leaf volatiles (GLVs) and terpenoids that play important roles in attracting natural enemies of phytophagous insects, including parasitic wasps [4]–[5], [7], [10]–[11]. Furthermore, recent studies using transgenic *Arabidopsis* plants show that genetic engineering of terpenoid metabolism, or overexpressing a single gene of terpenoid biosynthesis enables their ability to mediate the indirect defense of plants against spider mites or lepidopteran insects [12]–[13]. However, the chemical cues used by parasitoids are often composed of complex blends of herbivore-induced volatiles, thus making it difficult to understand the role of specific compounds in their host location [13]–[15]. In the case of eclosion from pupae, parasitoids encounter different conditions, forcing them to make the right choice to locate the preferred habitat for successful feeding and reproduction. Semiochemicals have been proved to serve important roles at all stages in the host-searching process of parasitoids [16]. However, evidence that the primary attractants involved in this process are used for host location by parasitoids, is very limited.

Agromyzid flies (Diptera: Agromyzidae) are exclusive plant feeders and best known as leafminers in host plants within diverse plant communities [17]. Most of agromyzids studied thus far have shown a very narrow range of host plant selection, i.e., only five species, and are considered truly polyphagous [17]–[18]. One of them, the pea leafminer, *Liriomyza huidobrensis* (Blanchard), has reportedly invaded almost all zoogeographical regions, rapidly extending its host range [18]–[19]. Among those host plants, the pea leafminer favors several families, including Fabaceae, Solanaceae and Cucurbitaceae [19]. To date, plant response to herbivorous infestation and the subsequent behavioral responses of natural enemies to these herbivores have focused primarily on leaf chewing [5], [10] and cell-content feeding insects [20]–[21], with a few cases for sucking/piercing insects [22]. The plant defense against the pea leafminer has been reported only for *Phaseolus vulgaris* in Fabaceae [23].

The present study includes cluster analyses of plant volatile profiles from seven families of host and non-host plants of pea leafminer and behavioral responses of a naive parasitic wasp, *Opius dissitus*, to some principal plant volatiles. We aim at understanding the potential evolutionary trends between herbivore-induced host plant volatiles and the host location of their parasitoids. Our findings include: 1) volatile profiles of eight host plants as responses to attacks by the polyphagous pea leafminer and the volatile profiles of two non-host plants as responses to JA treatment; 2) cluster analyses using these plant defense signals and inferring their evolutionary trend; 3) the involvement of indirect defenses to recruit parasitoids via induced volatiles emitted from selected host and non-host plants; 4) the behavioral responses of parasitoids to the odors of the host plant and non-host plant of the leafminer. Our results suggest that, as the most passive scenario of plant involvement, leafminers and mechanical damages evoke similar semio-chemicals. Using ubiquitous compounds, such as hexenol, for host location by general parasitoids could be an adaptation of the most conservative evolution of tritrophic interaction.

## Results and Discussion

Most of the published studies on induced indirect plant defense have thus far focused on tritrophic systems with one prey, i.e., one specific host plant and a specific predator/parasitoid [24]. In nature, herbivore usually deals with multiple predators and plants, and more complicated trophic influences [25]. For instance, a polyphagous spider mite, *Tetranychus urticae*, feeding on plants from various families could induce qualitative and quantitative variation among volatile profiles and the mite-induced volatile profiles differed qualitatively or quantitatively from the profile emitted from mechanically damaged or healthy plants [26]. However, very little has been studied about multiple host plants against polyphagous leafminer insects and their interactions with a parasitoid. The polyphagous pea leafminer has been known to feed on 14 plant species [17]. Our previous studies have demonstrated that *Opius dissitus* (a general parasitoid insect) is attracted to several leafminer-induced plant volatile compounds [7]. The present paper examines the indirect defense of host responses to the polyphagous leafminer, as well as elicited behavioral responses of parasitic wasps to those induced volatiles.

### Volatile profiles from healthy plants revealed a partly phylogenetic signal, while the inducible compounds of the infested-plants did not

The results of volatile analyses demonstrated that more than 90 constitutive and inducible compounds from volatile blends have been identified from host and non-host plants (see Table S1, 2, 3, 4). Healthy plant species released a few number of volatile compounds in lower concentrations in the families of Fabaceae, Solanaceae (species *Capsicum annuum*), Cucurbitaceae, Apiaceae, Rosaceae, and Vitaceae. In contrast, undamaged plants from Asteraceae and Solanaceae (species *Solanum lycopersicum*) constructively released many monoterpenes and sesquiterpenes in larger concentrations. Mechanically damaged plants emitted six-carbon alcohols, aldehydes, and esters, so-called green leaf voaltiles (GLVs), as dominant compounds in the blends except celery (*Apiumgraveolens*) and marigold (*Calendula officinalis*). Leafminer larvae-infested leaves and jasmonic acid (JA)- treated leaves from most plant families showed abundant productions of GLVs, monoterpenes, homoterpene, and sesquiterpenes, such as (*E*)-2-hexenal, (*Z*)-3-hexenol and (Z)-3-hexenyl acetate, ocimene, linalool, (3*E*)-4,8-dimethyl-1,3,7–nonatriene (DMNT), (3*E*,7*E*)-4,8,12-trimethyl-1,3,7,11-tridecatetraene (TMTT), caryophyllene and etc. Interestingly, when leafminer larvae-infested leaves or JA-treated leaves were compared to mechanically damaged leaves, we found that all the investigated plant species released one or more newly produced compounds. These results indicated that several biosynthetic pathways were induced by different treatments from the plants, some of which are very common biosynthetic pathways to produce the similar compounds and some of which are less common biosynthetic pathways to generate the specific compounds [8], [27].

**Figure 1:**
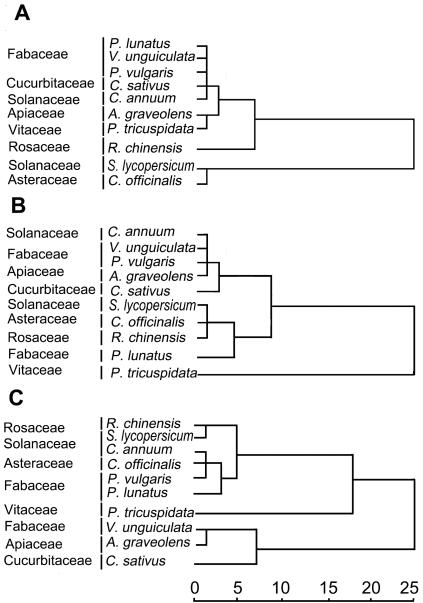
Hierarchical cluster analysis on the homogeneity using between-group-linkage method and square Euclidean distances based on the characters of total amounts and numbers of volatile compounds from: 1A, healthy plants; 1B, mechanically damaged plants; and 1C, leafminer-damaged host or JA-treated non-host plants of pea leafminer. The X axis is the relative distance between plants of seven plant families.

Hierarchical cluster analysis with association of two characters, i.e. the total amount and the numbers of volatile compounds emitted from healthy plants shows that the species of plants included in this analysis were categorically grouped into two clades, Solanaceae/Asteraceae (asterids) and the other families (predominantly rosids) on the second cluster branch (Fig. 1A)–with some similarity to the angiosperm phylogeny inferred from molecular and/or non-molecular data [28]–[29]. This suggested that the volatile compounds from healthy plants contains a signal of their intrinsic relationship in phylogeny. For instance, the three bean plant species (Fabaceae) were all found in the same polytomous clade. The only species of Cucurbitaceae was grouped in the same clade as the three Fabaceae. Indeed, recent publications on rosids show a close alliance of the order Fabales with the order Cucurbitales, both placed in one of the two subclades of eurosids I [29]–[30].

The only rosid species other than eurosids I included in this analysis is *Parhtenocissus tricuspidata* (Vitaceae), which is placed in a basal grade of rosids [28], [30]–[31] near the root of the large asterid clade. The basal position of Vitaceae is somewhat reflected in our smaller analysis, as Vitaceae are placed in a sister-clade to the aforementioned Fabaceae-Cucurbitaceae-clade. However, this picture is relativized by three groupings in the cladogram: 1) *Capsicum annuum* (Solanaceae) does not cluster with the other Solanaceae-species (*Solanum lycopersicum*) but is nested in the aforementioned Fabaceae-Cucurbitaceae-clade; 2) the representative of Apiaceae (member of euasterids II, sensu [30]) is also placed in the predominantly rosid clade of this analysis, as sister to the representative of Vitaceae; and 3) The representative of Rosaceae is placed outside of the remaining eurosids I plus Vitaceae-Apiaceae (Fig. 1A). Rosales to our current knowledge is placed in eurosids I, thus, the phylogenetic signal inferred from the position of Rosaceae in our current analysis would have been stronger if this family had been placed in the same clade as the three Fabaceae and the representative of Cucurbitaceae. Another phylogenetic signal of healthy plant volatiles is contained in the association of *Solanum lycopersicum* (Solanaceae) with *Calendula officinalis* (Asteraceae) in a clade deeply separated from the remaining taxa (Fig. 1a). Solanaceae (euasterids I) and Asteraceae (euasterids II) are both placed in euasterids in recent molecular analyses [29]–[30].

Concluding the findings in our cluster tree, we find a relatively good phlyogenetic resolution given the fact that only two characters were used (Fig. 1A). From many recent cladistic analyses it is known that only very few non-molecular or phytochemical characters are non-homoplasious [28]. To see whether numerical characters from healthy plant leaf volatiles contain really valuable phylogenetic information, however, more taxa would have to be included in an analysis. The number of taxa in our current tree was limited by the time resources. As for the inclusion of qualitative phytochemical characters from volatile plant compounds, the search in the comprehensive phytochemical literature [32]–[33] has revealed that most volatile plant compounds as identidfied with GC/MS are very homoplasious on a superfamilial level. For instance, the presence of monoterpenes could not be used as characters in the combined molecular and non-molecular analysis [28]. Also, most sesquiterpenes are very homplasious on a higher taxonomic level (with the exception of very few compounds, such as drimanes which occur only rarely outside the Winterales-clade in green plants). This feature of plant volatiles contrasts to the higher degree of non-homoplasy in other secondary compound classes, such as benzylisoquinoline alkaloids or ellagitannins [34]. Generally speaking, secondary compounds of higher molecular weight and structural complexity mostly contain more phylogenetic information. The current study, however, shows that the inclusion of leaf volatile quantitative characters could also be used for phylogenetic analyses and we encourage future work on this topic.

The cluster tree that was generated based on volatile profiles from damaged plants differed significantly from classic studies in angiosperms (Fig. 1 B, C). For example, three Fabaceae species were categorized into two separate clades and the rosids and asterids species crossed in the tree. These results indicate that the volatiles from damaged plants cannot be used to trace the relationship in their origins. This is partially explainable by the fact that the induced plant volatiles dominate most of the volatile blends of the host and non-host plants of the leafminer (see Table S1, 2, 3, 4), which maybe originated from the same precursor, such as octadecanoid pathway derived green leaf volatiles or terpenoids [27]. These volatile compounds are considered signals from indirect plant defenses that attract predators or parasitoids to attack feeding herbivores [4]–[5], [7], [10], [20], [25]. In a previous study involving the system of the vegetable leafminer, *L. sativae*, and its parasitoid, *Diglyphus isaea,* we found that neither the healthy host nor non-host plants of the leafminer elicited distinctive EAG responses in the parasitoid [35]. Odors of physically damaged leaves, whether host or non-host plants, elicited strong electroantennogram (EAG) responses of the leafminer and its parasitoid [35]. Therefore, we postulate that the indirect plant defense represents a common origin and universal evolutionary lineage.

### The induced volatile compound, (*Z*)-3-hexenol, as the primary damage attractant for the host location by the leafminer parasitoid

**Figure 2:**
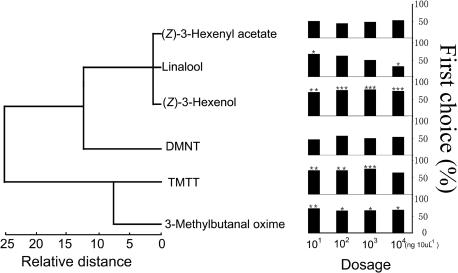
Six induced plant volatile compounds were analyzed by hierarchical cluster analysis using between-group-linkage method and square Euclidean distances, based on the probability of the induced volatile compound to be emitted by 10 host and non-host plants (left), and the behavioral responses (first choice %) of naive female *O. dissitus* to these six induced plant volatiles versus blank control (hexane) in Y-tube olfactometer at four dosages (right). DMNT: (3*E*)-4,8-dimethyl-1,3,7-nonatriene; TMTT: (3*E*,7*E*)-4,8,12-trimethyl-1,3,7,11-tridecatetraene. χ^2^ test for significant differences between numbers of parasitoids in each arm. * *P<*0.05; ** *P<*0.01; *** *P<*0.001. Forty to 45 females have made a choice in each dose. Unsuccessful parasitic wasp response rates in these experiments ranged from 10% to 25%.

It is widely known that parasitoid insects use induced volatile compounds from herbivore-damaged plants to locate their hosts [10], [36]–[37]. We have previously demonstrated that only six volatiles emitted from leafminer-damaged bean plants elicited either EAG responses or behavioral responses of a generalist parasitoid, *O. dissitus*, in an olfactometer bioassay [7]. (*Z*)-3-Hexenol, (Z)-3-hexenyl acetate, linalool, and (3*E*)-4,8-dimethyl-1,3,7–nonatriene (DMNT) appear to be the most homogeneous group with 70% of presence, while the induced compounds (3*E*,7*E*)-4,8,12-trimethyl-1,3,7,11-tridecatetraene (TMTT) and 3-methylbutanal oxime show a lesser degree of heterogeneity found only from a fewer host and non-host plants (less than 35% of abundance) (Fig. 2, left).

In the one-choice test, four of the six aforementioned compounds elicited positive responses from parasitic wasps, whereas (Z)-3-hexenyl acetate and DMNT did not (Fig. 2, right). Significant differences in the responses of parasitic wasps to (*Z*)-3-hexenol, TMTT and 3-methylbutanal oxime are observed at the different dosages we tested. These results may suggest that these three induced compound play the most important roles in host location of this parasitoid and the concentration of them is not a critical factor for attracting the parasitoids. However, the repellent activity of linalool was observed while the tested dosage increased 1000-fold.

**Figure 3:**
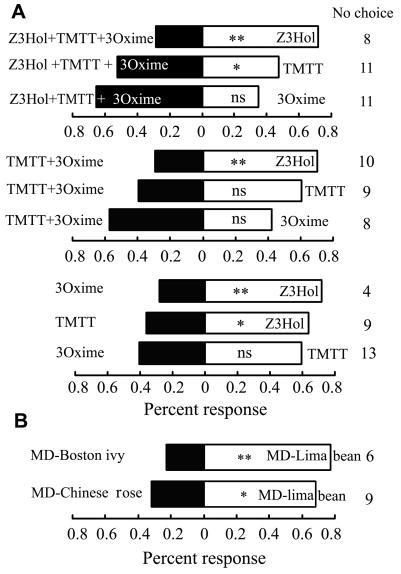
Responses of naive female *O. dissitus* in Y-olfactometer to different odor sources. 3A, responses to blends of compounds of authentic standards or individual compounds versus individual compounds. A three-compound mixture containing 50 ng 10 µL^−1^ of each Z3Hol: (Z)-3-hexenol, TMTT: (3*E*,7*E*)-4,8,12-trimethyl-1,3,7,11-tridecatetraene, and 3Oxime: 3-methylbutanal oxime; and a two-compound mixture containing 75 ng 10 µL^−1^ of TMTT and 3-methylbutanal oxime, while each individual compound is diluted to the concentration of 150 ng per 10 microliters. 3B, responses to the odors of mechanically damaged (MD) host plant (lima beans) and mechanically damaged (MD) non-host plants (Chinese rose and Boston ivy). χ^2^ test for significant differences between numbers of parasitoids in each arm. * *P<*0.05; ** *P<*0.01. Forty to 45 females have made a choice in each experiment.

Further dual-selection experiments to compare the individual induced-compounds to their blend of mixtures showed that the parasitic wasp responded preferably to (*Z*)-3-hexenol to either mixture blends or individual compounds (Fig. 3A). The observed higher percentages of parasitoid responses to the mixture blend than to 3-methylbutanal oxime could also be due to the effect of their strong attraction to one of blend compounds, (*Z*)-3-hexenol. This may also suggest that the naive parasitoid uses (*Z*)-3-hexenol from mechanically- or leafminer-damaged plants as the primary damage attractant to locate the prey/host plant because this compound with its high volatility may be the first sensed by parasitoids. In a previous study on the *Phaseolus vulgaris*-*Liriomyza spp.-Opius dissitus* tritrophic system, we hypothesized that the predominant compounds in herbivore-induced volatile blends play an important role in mediating parasitoid search behavior over relatively long distances, while secondary and minor compounds improve the precision of host location over short distances [7]. In present study, the single- and dual-choice data clearly indicated that (*Z*)-3-hexenol is the more important general damage attractant, while TMTT and 3-oxime are the important distinguishing attractants.

Hoballah *et al.* [11] suggested that the higher volatility of some green leaf compounds may play a critical role in the initial attraction of naive natural enemies to damaged plants. Interestingly, it has been reported that generalist parasitoids of lepidopteran larvae are more attracted to caterpillar-induced GLVs than to induced terpenoids, such as DMNT, and TMNT, etc. [38]. Some novel approaches to the study of the attractiveness of herbivore-induced plant volatiles to parasitoids further showed that a naive generalist parasitoid, *Cotesia marginiventris*, prefers partially altered volatile blends or blends of freshly damaged plants containing high amounts of green-leaf volatiles to those with high amounts of sesquiterpenes [14]–[15]. In the present study, (*Z*)-3-hexenol has been identified in all plants except celery, providing further evidence that this structural uniformity due to the activation of a common set of biosynthetic pathways was shared by a wide range of plants and might be used by a broad spectrum of insect parasitoids and predators. The widely distributed (*Z*)-3-hexenol from plants initiating the host location of leafminer parasitoid may indicate a parsimonious scenario in the evolution of plant defense.

### Ability of Parasitoids to distinguish the host plant from non-host plant of the leafminer

(*Z*)-3-hexenol appears to predominate in volatile blends collected almost in all plants immediately after wounding [11], [39]( see Table S1, 2, 3, 4). Our previous study showed that the vegetable leafminer, *L. sativae*, has higher EAG responses to synthetic C_6_ GLVs including (*Z*)-3-hexenol [40]. Bruce *et al.* [41] showed that phytophagous insects also use this compound plus other GLVs and terpenoids to form a ratio-specific odor for host plants location. Therefore, this volatile presents an important cue for herbivores and their natural enemies. However, the question that needs to be addressed is whether the parasitoid can precisely recognize the leafminer host plants and the non-host plants by relying solely on the (*Z*)-3-hexenol cue. Our study compared the attraction of the parasitoid to a host plant (lima beans) and non-host plants (Chinese rose and Boston ivy) focusing exclusively on odor cues (Fig. 3B). The volatile analysis showed that mechanically damaged lima beans released larger amounts of (*Z*)-3-hexenol than the rose, but lower amounts than the Boston ivy (Fig. S1A). However, the concentration of the (*Z*)-3-hexenol is not a critical factor for attracting the parasitoids (Fig. 2, right). Lima beans were preferred over the two non-host plant odors by the parasitoids (Fig. 3B; bean vs rose: χ^2^ = 5.54, *P*<0.05, n = 41; bean vs Boston ivy: χ^2^ = 13.09, *P*<0.001, n = 44), indicating that parasitoids were able to distinguish the leafminer's host plants from non-host plants based not only on this universal compound but also on other chemical cues involved (Fig. S1 and Table S4). The result is very consistent with the reports on cowpea and maize (comparison by Hoballah *et al.* [11]). Another novel study on spider mites showed that the volatile blends were similar for most components between spider mite-damaged and JA-treated lima bean, whereas spider-mite induced odor was more attractive to predatory mites which was due to the difference in methyl salicylate (MeSA) [21]. In the present study, the attractants in the blends of host and non-host plants have been identified and compared (Fig. S1). Therefore, another possible scenario we postulated is that any antagonistic volatile compounds within the blends of non-host plants may disorient the search path of parasitoids to non-host plant of the leafminers.

Our results suggest that the most passive scenario of plant involvement is that leafminers and mechanical damages evoke similar semio-chemicals. This scenario has been verified in several plant families such as Cucurbitaceae, Fabaceae, Solanaceae, Ginkgoaceae, and Vitaceae [25]–[26]. Using ubiquitous compounds, such as green leaf volatiles, for host location by general parasitoids could be an adaptation of the most conservative evolution of tritrophic interaction. Although (*Z*)-3-hexenol plays an important role in the initial step of parasitoid host location, other specifically induced volatiles may be used by some predatory insects/parasitoids and the capability of associated learning of parasitoids are also essential in this process [5], [10], [14]–[15]. Some secondary metabolites or compounds may be used to improve the precision of host location, or involve other host location cues, such as visual, contact and taste cue [36].

## Materials and Methods

### Plant materials

Seeds of the tested host plants were planted in individual plastic pots of 12 cm diameter containing a mixture of peat and vermiculite (4∶1). All plants were grown in environmental chambers (Conviron Co., Winnipeg, Manitoba, Canada) under 25°C (±5°C), 14-h∶10-h light∶dark (L∶D) photoperiod and 60±10% R.H. Fabaceae plants (bean, *Phaseolus vulgaris*; lima bean, *P. lunatus;* and cowpea, *Vigna unguiculata*) were used at growth ages of 2–3 weeks. Bell pepper (*Capsicum annuum*), tomato (*Solanum lycopersicum*), cucumber (*Cucumins sativus*), marigold (*Calendula officinalis*) and celery (*Apium graveolens*) were used at an age of 6–8 weeks. The non-host plants—Chinese Rose (*Rosa chinensis*) and Boston ivy (*Parthenocissus tricuspidata*)—were collected locally at the age of around 6 weeks. Plant origins are listed in EMS Table S5 in Supplementary Material.

### Insects

Two-week-old kidney bean plants with two fully developed true leaves were used to cultivate leafminers. The *Opius dissitus* parasitoids were reared on colonies of *L. huidobrensis* that were fed on kidney bean plants. *O. dissitus* and *L. huidobrensis* cultures were maintained in separate environmental chambers (Donglian Co., Harbing, China) at 25°C (±1°C), 70±10% RH, with a 14 h∶10h (L∶D) photoperiod. Females of *O. dissitus* emerged from pupae in glass tubes (70×8 mm) and were mated within 24 h. They were kept in glass vials (80×23 mm) with a supply of a honey solution (10%) under the same environmental conditions as described above. All *O. dissitus* used in the behavioral assays were two- to four-day-old adult females with no previous exposure to their host, *L. huidobrensis,* or host plants. Each was used only once in the experiments. Cultures of *O. dissitus* had been propagated for three years under these laboratory conditions.

### Plant treatments

Plants used to collect volatiles were treated similar to methods described by Wei *et al.* [23]. In most cases, healthy plants were exposed to 150–200 adult pea leafminers for 2 h so that they obtained a cohort of second instars larvae after 4 to 5 days. In order to mimic the damage caused by pea leafminer in non-host plants, we used a method similar to the one described by Dicke *et al.* [21]. One Chinese rose shoot plant with 4–6 sets of leaves or a Boston ivy shoot plant with 15–20 leaves was placed with stems into vials containing 6 mL of an aqueous jasmonic acid (JA) (Sigma-Aldrich Co., St. Louis, Missouri, USA) solution (1 mM) for 36 h [42]. JA has been dissolved in 0.5% ethanol (Beijing Huateng Chemical Co., Ltd., Beijing). The vials were sealed with parafilm. The control plants were placed in vials with 6 mL of 0.5% alcohol for 36 h. Before volatile collections, the JA-treated plants and the controls were transferred from their vials to individual glass tubes filled with 50 mL of tap water. Half of the control shoots were used for collection as the treatment for undamaged leaves, and the other half of the leaves were cut with a blade as treatment for mechanically damaged leaves (200 cuts per plant). All were subjected to the volatile collection system.

### Plant volatile collection

With minor modification, the design for the headspace volatile collection system was similar to that described by Wei *et al.* [23]. Three potted host plants or cut shoots of non-host plants were -placed in a plastic oven bag (40×44 cm, Reynolds^®^, US, with an approximate 7500 mL in volume). The collection bag was sealed around each stem approximately 4–5 cm above soil surface or over the vial containing JA-treated non-host plants. Using two freshly activated charcoal traps, a stream of filtered and moisturized air was pumped into the bag. The air with emitted plant volatiles was withdrawn through a glass collector by a membrane pump (Beijing Institute of Labor Instruments, China) at a rate of 400 mL min^−1^. The absorbing glass collector contained 100 mg of Porapak Q (80–100 mesh size, Supelco, USA), and headspace was collected for 10 h. Five collections were made simultaneously. The first four bags contained three potted plants each; the fifth bag, which served as the control, had no plants. The absorbed volatile compounds from the Porapak Q collectors were then extracted with 700 µL of HPLC-grade dichloromethane (Tedia Company, USA). All aeration extracts were stored at −20°C until used in chemical analyses or behavioral experiments. Plants were weighed immediately after collections using an electronic balance (Mettler AE 240, Shanghai, China). Numbers of larvae on the leaves of leafminer infested plants were recorded by carefully examining leaves under a stereo microscope (Wild, Heerbrugg, Switzerland).

### Chemical identification and quantification of collected volatiles

The collected volatile compounds were identified using an Agilent gas chromatographer (GC) (6890N) coupled with a mass spectrometry (MS) system (5973 MSD, Agilent Technologies, Inc. USA). The system was equipped with either a DB-WAX polyethylene glycol 20000 column (60 m×0.25 mm ID, 0.15-µm film thickness), or a DB5-MS column (95% polydimethyl siloxae 5% poly-1,4-bis-dimethylsiloxae phenylene siloxae, 60 m × 0.25 mm ID×0.15-µm film thickness, Agilent Technologies, Palo Alto, CA, USA). For analyses using the DB-WAX column, the initial oven temperature was kept at 40°C for 4 min and then increased to 180°C at a programmed rate of 5°C min^−1^, followed by a rate of 10°C min^−1^ to 230°C. On a DB5-MS column, the GC oven temperature was kept at 40°C for 4 min and then increased to 200°C at a rate of 5°C min^−1^, followed by a rate at 20°C min^−1^ to 280°C. The inlet was operated under the splitless injection mode, and the injector temperature was maintained at 250°C with a constant flow rate at 1.0 mL min^−1^. The GC-MS electron impact source was operated in the scan mode with the MS source temperature at 230°C and the MS Quad at 150°C. Heptanoic acid, ethyl ester and dodecanoic acid, ethyl ester (1 ng µL^−1^, 5 ng µL^−1^, 20 ng µL^−1^, 50 ng µL^−1^, 100 ng µL^−1^) were used as external standards for developing standard curves to quantify volatiles in the samples. Volatile compounds were identified by comparing their retention times and spectra with those of synthetic standards (see details in [23] and Table S1, 2, 3, 4). Referenced mass spectra from the NIST02 library (Scientific Instrument Services, Inc., USA) were also used.

### Behavioral bioassay

A Y-tube olfactometer was used to investigate the behavioral responses of female *O. dissitus* (parasitic wasp) to synthetic compounds of individual induced volatiles or a blend of mixtures from the host or non-host plants [7]. Each female parasitic wasp was placed in the olfactometer for 5 min. A “no choice” was recorded when the wasp remained inactive for the duration of the testing period. A “first choice” was declared whenever the wasp moved more than 5 cm into either arm (visually assessed by a line marked on both arms). Previous tests had shown that female *O. dissitus* had no preference for either solvent control (dichloromethane or hexane) [7].

The selected single compounds, either those that elicited EAG active responses from the parasitic wasp or those that evoked positive behavioral responses [7], were diluted with HPLC-grade hexane in a grade of 10 ng, 100 ng, 1000 ng, and 10000 ng per 10 microliters. Each tested compound at the aforementioned dosages was applied to a piece of filter paper (1×2 cm) at a volume of 10 microliters and was placed inside one arm of the Y-tube olfactometer. A same-sized filter paper impregnated with equal volume of HPLC-grade hexane was set in the other arm as the control. In the dual-choice tests, either a three-compound mixture containing 50 ng 10 µL^−1^ of each (Z)-3-hexenol, TMTT, and 3-methylbutanal oxime (generously donated by Dr R. Kaiser of Givaudan Schweiz AG, Dubendorf, Switzerland; a mixture of syn and anti- isomers in a ratio of 1: 1; purity by gas-chromatography is at least 95%), or a two-compound mixture containing 75 ng 10 µL^−1^ of TMTT and 3-methylbutanal oxime, or a single compound in a concentration of 150 ng 10 µL^−1^ paired with each individual compound containing 150 ng 10 µL^−1^ were tested. Filter paper with individual compounds (10 µL) was placed in one arm, and the other treatment was set in the other arm. For *O. dissitus* females to choose the extracts from mechanically damaged host or non-host plants, the mean dosage of each blend used was equivalent to 0.2 h entrainment of volatiles. Filter papers with the chemical compounds or hexane control were refreshed after each test.

### Data analysis

Data were analyzed using the SPSS statistical program (version 11.0; SPSS Inc., USA). Student's *t*-test or analysis of variance (ANOVA) and Turkey's honestly significant difference (HSD) test were used to compare volatile emissions from different plant treatments. Each experiment was replicated four to six times. The relative percentage of volatile compounds was arcsine (x^1/2^) transformed, whereas absolute quantities of emitted volatile compounds were log (x+1) transformed to correct for heterogeneity of variances before statistical analysis. A χ^2^ test was used to determine the significance of the differences between the numbers of parasitoids choosing each arm of the olfactometer [7]. Parasitoids that did not make a choice were excluded from statistical analyses. Unsuccessful parasitic wasp response rates in choice experiments ranged from 10% to 25%.

The homogeneity of total amounts (absolute quantity of volatiles detected in a blend) and numbers of volatile compounds (see Table S 1, 2, 3, 4) from healthy and damaged plants were analyzed by hierarchical cluster analysis using a between-group-linkage method and square Euclidean distances (SPSS, version 11.0). These two characters indicate the traits of the volatile emission of a plant. Volatiles present at 0.1% or higher proportions in the headspace samples are subjected to cluster analysis. Six EAG-responded volatiles [7] were also subjected to hierarchical cluster analysis using the same method, which was based on the probability that the induced volatile compound appeared from emitted volatiles of mechanically- and leafminer-damaged host plants or JA-treated non-host plants. Due to the fact that accumulated data were also variables to normalize, these data were transformed (Z-scores) and standardized to obtain normally distributed variables.

## Supporting Information

Table S1 Relative amounts of volatiles released from 3 fabaceous plants with undamaged leaf (UL), mechanically damaged leaf with a blade (MDL), and *L. huidobrensis* larvae-damaged leaf (Lh-LDL).
(0.12 MB DOC)

Table S2 Relative amount of volatiles released from Solanaceae plants by undamaged leaf (UL), mechanically damaged leaf with a blade (MDL), and *L. huidobrensis* larvae-damaged leaf (Lh-LDL).
(0.12 MB DOC)

Table S3 Relative amount of volatiles released from Cucurbitaceae, Apiaceae, Asteraceae plants by undamaged leaf (UL), mechanically damaged leaf with a blade (MDL), and *L. huidobrensis* larvae-damaged leaf (Lh-LDL).
(0.10 MB DOC)

Table S4 Relative amount of volatiles released from Rosaceae and Vitaceae plants by undamaged leaf (UL), mechanically damaged leaf with a blade (MDL), and JA-treated leaf
(0.12 MB DOC)

Table S5 Plants used in the headspace experiments.
(0.04 MB DOC)

Figure S1 Absolute amounts of 6 principal induced volatile compounds from 1A, headspace collections of mechanically damaged plants, and from 1B, leafminer-damaged host plants or JA-treated non-host plants. The amounts of volatile released from JA-treated plants were expressed as nanogram per 10 gram fresh weight (FW) per 1h.
(1.20 MB DOC)
