# Supplementary material for: Plants Attract Parasitic Wasps to Defend Themselves against Insect Pests by Releasing Hexenol
Source: PLoS One. 2007 Sep 5;2(9):e852. doi: 10.1371/journal.pone.0000852 (PMC1955833; doi:10.1371/journal.pone.0000852)
Supplement: Table S1 — Relative amounts of volatiles released from 3 fabaceous plants with undamaged leaf (UL), mechanically damaged leaf with a blade (MDL), and L. huidobrensis larvae-damaged leaf (Lh-LDL). (0.12 MB DOC) [file pone.0000852.s001.doc]

| **Table S1** Relative amounts of volatiles released from 3 fabaceous plants with undamaged leaf (UL), mechanically damaged leaf with a blade (MDL), and *L. huidobrensis* larvae-damaged leaf (Lh-LDL). | | | | | | | | | |
| --- | --- | --- | --- | --- | --- | --- | --- | --- | --- |
| **Chemical compound ^*^** | **Fabaceae** | | | | | | | | |
|  | ***V. unguiculata*** ***P. lunatus******P. vulgaris*** ^║^ | | | | | | | | |
|  | Relative % of compounds in different treatments (means ± SE) | | | | | | | | |
|  | UL | MDL | Lh-LDL | UL | MDL | Lh-LDL | UL^║^ | MDL^║^ | Lh-LDL^║^ |
| **Green leaf volatiles** |  |  |  |  |  |  |  |  |  |
| (*Z*)-3-hexenyl butyrate **^†^** | ― | ― | ― | ― | 1.6±0.1 | 0.2±0.05 | ― | ― | 1.3±0.5 |
| (*Z*)-3-hexenyl iso-valerate **^†^** | ― | ― | ― | ― | 1.3±0.1 | 0.5±0.1 | ― | ― | 0.8±0.1 |
| 1-hexyl acetate **^†^** | ― | 1.9±0.2 | 1.3±0.2 | ― | 0.4±0.1 | 0.4±0.1 | ― | ― | ― |
| 1-octanol **^†^** | ― | ― | ― | ― | 0.7±0.1 | 0.2±0.1 | ― | ― | ― |
| 1-pentanol | ― | ― | ― | ― | ― | ― | ― | ― | 0.1±0.02 |
| 1-octen-3-ol | ― | 7.8±0.4 | 0.9±0.3 | 39.5±7.4 | 8.5±1.0 | 1.3±0.9 | ― | 2.3±0.2 | 0.3±0.1 |
| 3-octanol **^†^** | ― | ― | ― | ― | 0.9±0.03 | ― | ― | ― | ― |
| (*E*)-2-hexenol | ― | ― | ― | ― | 0.6±0.3 | ― | ― | ― | ― |
| hexenyl acetate **^†^** | ― | ― | ― | ― | 14.0±1.7 | 14.2±1.3 | ― | ― | ― |
| (*E*)-2-hexenyl acetate **^†^** | ― | ― | ― | ― | 0.3±0.1 | 0.5±0.3 | ― | ― | ― |
| hexenol | ― | ― | ― | ― | 2.7±0.04 | 0.5±0.3 | ― | ― | ― |
| (*E*)-3-hexenol **^†^** | ― | ― | ― | ― | 0.4±0.1 | ― | ― | ― | ― |
| hexanal | ― | ― | ― | ― | ― | ― | ― | 4.7±1.2 | 1.3±0.5 |
| hexenal **^†^** | ― | ― | ― | ― | 0.7±0.1 | 0.5±0.2 | ― | ― | ― |
| (*Z*)-3-hexenal **^†^** | ― | ― | ― | ― | 4.2±2.2 | 0.9±0.3 | ― | ― | ― |
| (*E*)-2-hexenal | ― | ― | ― | ― | 3.9±1.5 | 0.3±0.2 | ― | 5.8±0.4 | 0.2±0.1 |
| (*Z*)-3-hexenyl acetate | ― | 61.2±2.6 | 4.6±0.7 | ― | ― | ― | 37.3±8.5 | 34.0±8.5 | 21.5±5.4 |
| (*Z*)-3-hexenyl iso-butyrate **^†^** | ― | ― | ― | ― | 0.2±0.02 | 0.2±0.03 | ― | ― | ― |
| (*Z*)-3-hexenol | ― | 12.3±2.3 | 1.1±0.2 | ― | 46.4±0.4 | 6.6±3.8 | ― | 20.3 ± 3.1 | 7.8±4.6 |
| **Terpenoid** |  |  |  |  |  |  |  |  |  |
| nerolidol **^†^** | ― | ― | ― | ― | ― | 0.2±0.1 | ― | ― | ― |
| (*Z*)-*β*-farnesene | ― | ― | 7.3±0.7 | ― | ― | ― | ― | ― | ― |
| limonene | 100.0 | 3.4±0.3 | 3.2±0.3 | ― | ― | ― | ― | ― | ― |
| ocimene | ― | ― | ― | ― | ― | 0.2±0.03 | ― | ― | ― |
| (*E*)-*β*-ocimene | ― | 3.8±0.2 | 3.8±0.6 | ― | 2.7±0.9 | 18.9±4.9 | ― | 2.1 ± 0.5 | 1.0±0.2 |
| (*Z*)-*β*-ocimene | ― | ― | ― | ― | ― | 0.4±0.1 | ― | ― | ― |
| DMNT ^§^ | ― | 2.1±0.4 | 66.4±1.9 | ― | 3.5±1.0 | 15.5±0.6 | 62.7±8.5 | 5.1 ± 0.8 | 23.9±3.8 |
| linalool | ― | 1.2±0.1 | 0.8±0.2 | ― | 0.7±0.2 | 2.7±0.3 | ― | 4.6 ± 3.2 | 1.0±0.2 |
| *β*-caryophyllene | ― | 1.5±0.2 | 1.5±0.1 | ― | 0.2±0.1 | 0.9±0.1 | ― | ― | 3.2±0.4 |
| *α*-humulene | ― | ― | ― | ― | ― | ― | ― | ― | 0.3±0.03 |
| (*E*,*E*)-*α*-farnesene | ― | ― | ― | ― | ― | ― | ― | 0.9 ± 0.3 | 2.4±0.8 |
| TMTT ^¶^ | ― | ― | 2.2±0.3 | ― | 1.1±0.3 | 24.2±4.8 | ― | ― | 5.3±1.2 |
| **Oximes** |  |  |  |  |  |  |  |  |  |
| syn -2-methylpropanal oxime | ― | ― | 1.6±0.1 | ― | 0.2±0.1 | 3.3±0.6 | ― | 4.9 ± 1.5 | 8.2±1.7 |
| anti -2-methylpropanal oxime | ― | ― | ― | ― | 0.2±0.1 | 0.9±0.3 | ― | 2.1 ± 0.7 | 2.0±0.3 |
| syn -2-methylbutanal oxime | ― | ― | 2.6±0.5 | ― | 1.0±0.7 | 2.6±0.4 | ― | 4.2 ± 1.3 | 9.3±0.9 |
| anti -2-methylbutanal oxime | ― | ― | ― | ― | 0.1±0.03 | 0.6±0.1 | ― | ― | 2.8±0.3 |
| syn -3-methylbutanal oxime | ― | ― | ― | ― | ― | 0.2±0.02 | ― | ― | 1.0±0.2 |
| anti -3-methylbutanal oxime | ― | ― | ― | ― | ― | 0.3±0.1 | ― | ― | 0.6±0.3 |
| **Other compounds** |  |  |  |  |  |  |  |  |  |
| methyl salicylate | ― | ― | ― | ― | 0.3±0.03 | 0.3±0.1 | ― | ― | 0.5±0.1 |
| dodecane | ― | 4.8±0.7 | 2.7±0.3 | ― | ― | ― | ― | 7.8 ± 3.1 | 3.2±0.7 |
| 3-methylbutyl acetate **^†^** | ― ^‡^ | ― | ― | ― | ― | 1.0±0.2 | ― | ― | ― |
| 3-octanone | ― | ― | ― | ― | 0.9±0.1 | 0.4±0.2 | ― | 1.3 ± 0.4 | 0.1±0.03 |
| 2-cyclopentylcyclopentanone **^†^** | ― | ― | ― | 60.5±7.4 | 1.7±0.5 | 0.6±0.2 | ― | ― | ― |
| (*Z*)-2-pentenol **^†^** | ― | ― | ― | ― | 0.2±0.1 | 0.5±0.3 | ― | ― | ― |
| 2-methylbutanol **^†^** | ― | ― | ― | ― | 0.2±0.03 | ― | ― | ― | ― |
| 2-ethylcyclopentanone **^†^** | ― | ― | ― | ― | ― | ― | ― | ― | 1.9±0.6 |
| **Total number of chemicals** | **1** | **10** | **14** | **2** | **30** | **32** | **2** | **14** | **25** |

***** Volatiles present at 0.1% or higher proportions in the headspace samples are listed in the table.

^†^ Compounds were tentatively identified by comparison of their MS-spectra with those of in the NIST02 library (Scientific Instrument Services, Inc., USA).

^‡^ Compounds marked with “―” means under detectable level.

^§^ DMNT: (3*E*)-4,8-dimethyl-1,3,7–nonatriene.

^¶^ TMTT: (3*E*,7*E*)-4,8,12-trimethyl-1,3,7,11-tridecatetraene.

^║^ Data have been published as Wei *et al*.^1^.

.

# Wei, J-N., Zhu, J. & Kang, L. Volatiles released from bean plants in response to agromyzid flies. Planta **224**, 279-287 (2006).
