# Supplementary material for: Plants Attract Parasitic Wasps to Defend Themselves against Insect Pests by Releasing Hexenol
Source: PLoS One. 2007 Sep 5;2(9):e852. doi: 10.1371/journal.pone.0000852 (PMC1955833; doi:10.1371/journal.pone.0000852)
Supplement: Table S2 — Relative amount of volatiles released from Solanaceae plants by undamaged leaf (UL), mechanically damaged leaf with a blade (MDL), and L. huidobrensis larvae-damaged leaf (Lh-LDL). (0.12 MB DOC) [file pone.0000852.s002.doc]

| **Table S2** Relative amount of volatiles released from Solanaceae plants by undamaged leaf (UL), mechanically damaged leaf with a blade (MDL), and *L. huidobrensis* larvae-damaged leaf (Lh-LDL). | | | | | | |
| --- | --- | --- | --- | --- | --- | --- |
| **Chemical compound^*^** | **Solanaceae** | | | | | |
|  | ***S. lycopersicum C. annuum*** | | | | | |
|  | Relative % of compounds in different treatments (means ± SE) | | | | | |
|  | UL | MDL | Lh-LDL | UL | MDL | Lh-LDL |
| **Green leaf volatiles** |  |  |  |  |  |  |
| (*Z*)-3-hexenyl butyrate **^†^** | ―^‡^ | 0.4±0.1 | ― | ― | ― | ― |
| (*Z*)-3-hexenyl acetate | ― | 0.3±0.2 | ― | ― | 13.3±0.9 | 2.2±0.2 |
| (*Z*)-3-hexenol | ― | 4.9±2.3 | ― | ― | 43.4±3.3 | 4.5±0.1 |
| (*E*)-2-hexenal | ― | ― | ― | ― | 1.0±0.1 | ― |
| (*Z*)-3-hexenal **^†^** | ― | ― | ― | ― | 1.8±0.2 | ― |
| (*Z*)-3-hexenyl iso-valerate **^†^** | ― | ― | ― | ― | ― | 0.5±0.1 |
| **Terpenoid** |  |  |  |  |  |  |
| eremophilene **^†^** | ― | ― | ― | ― | ― | 0.5±0.2 |
| zingiberene **^†^** | ― | ― | ― | ― | ― | 0.3±0.05 |
| *β*-myrcene | ― | ― | ― | ― | ― | 0.3±0.01 |
| *β*-pinene | 0.1±0.01 | 0.3±0.03 | 0.1±0.004 | ― | ― | ― |
| *α*-pinene | 3.6±0.2 | 9.7±2.3 | 3.1±0.1 | ― | ― | ― |
| 2-carene **^†^** | 20.1±0.3 | 17.0±1.6 | 20.4±0.6 | ― | ― | ― |
| 3-carene | ― | 0.2±0.02 | ― | ― | ― | ― |
| *β*-cymene **^†^** | 2.4±0.3 | 2.6±0.2 | 2.9±0.2 | ― | ― | ― |
| sabinene **^†^** | 0.3±0.1 | 1.1±0.04 | 0.1±0.01 | ― | ― | ― |
| *α*-terpinene | 1.7±0.2 | 2.1±0.2 | 1.5±0.1 | ― | ― | ― |
| terpinolene | 0.4±0.03 | 0.3±0.01 | 0.3±0.02 | ― | ― | ― |
| δ-terpinene **^†^** | ― | ― | 0.2±0.01 | ― | ― | ― |
| γ-terpinene | 0.2±0.02 | 0.3±0.05 | ― | ― | ― | ― |
| *α*-muurolene **^†^** | ― | ― | ― | ― | ― | 2.4±0.9 |
| *α*-elemene **^†^** | ― | ― | ― | ― | ― | 0.5±0.1 |
| ε-elemene **^†^** | ― | 0.7±0.1 | 0.9±0.1 | ― | ― | ― |
| *β*-elemene † | 0.2±0.03 | 0.2±0.04 | 0.1±0.01 | ― | ― | 8.4±1.6 |
| δ-elemene **^†^** | 1.9±0.2 | ― | ― | ― | ― | ― |
| *α*-humulene | 0.5±0.03 | 0.7±0.1 | 0.5±0.03 | ― | ― | ― |
| germacrene D **^†^** | ― | 0.1±0.01 | ― | ― | ― | ― |
| *β*-phellandrene | 49.2±1.3 | 43.1±3.2 | 50.3±0.6 | ― | ― | ― |
| *α*-phellandrene | 4.4±0.3 | 3.3±0.1 | 4.1±0.2 | ― | ― | ― |
| limonene | 12.4±0.7 | 8.5±0.6 | 11.7±0.4 | ― | ― | 1.4±0.3 |
| (*Z*)- *β-*ocimene | ― | 0.3±0.01 | ― | ― | ― | 0.4±0.03 |
| (*E*)- *β-*ocimene | ― | ― | ― | ― | ― | 20.3±0.9 |
| *β-*caryophyllene | 2.6±0.1 | 4.1±0.7 | 2.6±0.2 | ― | ― | ― |
| (*E*,*Z*)-4,8,12-trimethyl-1,3,7,11-tridecatetraene **^†^** | ― | ― | ― | ― | ― | 0.4±0.1 |
| DMNT ^§^ | ― | ― | ― | 13.9±2.8 | 24.2±1.6 | 46.6±3.0 |
| TMTT ^¶^ | ― | ― | 0.9±0.4 | ― | ― | 2.8±0.4 |
| (*Z*)-4,8-dimethyl-1,3,7-nonatriene | ― | ― | ― | ― | 1.1±0.4 | 3.0±0.2 |
| linalool | ― | ― | ― | ― | 12.3±0.1 | 2.0±0.1 |
| (*E*,*E*)-*α*-farnesene | ― | ― | ― | ― | ― | 0.4±0.1 |
| (*E,Z*)-2,6-dimethyl-2,4,6-octatriene**^†^** | ― | ― | ― | ― | ― | ― |
| **Other compounds** |  |  |  |  |  |  |
| cis-jasmone | ― | ― | ― | ― | ― | 0.5±0.2 |
| 2-cyclopentylcyclopentanone **^†^** | ― | ― | ― | 70.0±2.4 | 2.2±0.04 | 1.1±0.2 |
| phenyl ethyl alcohol **^†^** | ― | ― | ― | ― | ― | 1.3±0.4 |
| 2-ethylcyclopentanone **^†^** | ― | ― | ― | 16.1±2.2 | 0.8±0.2 | ― |
| **Total number of chemicals** | **15** | **21** | **16** | **3** | **9** | **21** |

***** Volatiles present at 0.1% or higher proportions in the headspace samples are listed in the table.

^†^ Compounds were tentatively identified by comparison of their MS-spectra with those of in the NIST02 library (Scientific Instrument Services, Inc., USA).

^‡^ Compounds marked with “―” means under detectable level.

^§^ DMNT: (3*E*)-4,8-dimethyl-1,3,7-nonatriene.

^¶^ TMTT: (3*E*,7*E*)-4,8,12-trimethyl-1,3,7,11-tridecatetraene.
