# Supplementary material for: Plants Attract Parasitic Wasps to Defend Themselves against Insect Pests by Releasing Hexenol
Source: PLoS One. 2007 Sep 5;2(9):e852. doi: 10.1371/journal.pone.0000852 (PMC1955833; doi:10.1371/journal.pone.0000852)
Supplement: Table S3 — Relative amount of volatiles released from Cucurbitaceae, Apiaceae, Asteraceae plants by undamaged leaf (UL), mechanically damaged leaf with a blade (MDL), and L. huidobrensis larvae-damaged leaf (Lh-LDL). (0.10 MB DOC) [file pone.0000852.s003.doc]

| **Table S3** Relative amount of volatiles released from Cucurbitaceae, Apiaceae, Asteraceae plants by undamaged leaf (UL), mechanically damaged leaf with a blade (MDL), and *L. huidobrensis* larvae-damaged leaf (Lh-LDL). | | | | | | | | | |
| --- | --- | --- | --- | --- | --- | --- | --- | --- | --- |
| **Chemical compound^*^** | **Cucurbitaceae Apiaceae Asteraceae** | | | | | | | | |
|  | ***C. sativus A. graveolens C. officinalis*** | | | | | | | | |
|  | Relative % of compounds in different treatments (means ± SE) | | | | | | | | |
|  | UL | MDL | Lh-LDL | UL | MDL | Lh-LDL | UL | MDL | Lh-LDL |
| **Green leaf volatiles** |  |  |  |  |  |  |  |  |  |
| (*Z*)-3-hexenyl acetate | ―^‡^ | 41.5±5.7 | 9.9±2.5 | ― | ― | ― | ― | ― | ― |
| (*Z*)-3-hexenol | ― | 48.4±3.9 | 4.1±0.9 | ― | ― | ― | 0.5±0.3 | 1.6±0.6 | 0.3±0.1 |
| 1-octanol **^†^** | ― | ― | ― | ― | ― | ― | 0.5±0.04 | 0.2±0.01 | 0.8±0.3 |
| **Terpenoid** |  |  |  |  |  |  |  |  |  |
| *β*-myrcene | ― | ― | ― | ― | 2.2±0.4 | 1.6±0.04 | ― | ― | ― |
| *β*-pinene | ― | ― | ― | ― | 0.4±0.2 | ― | 1.1±0.1 | 0.8±0.1 | 0.8±0.1 |
| *α*-pinene | ― | ― | ― | ― | ― | ― | 22.4±2.3 | 28.7±1.4 | 25.6±1.0 |
| 3- carene | ― | ― | ― | ― | ― | ― | 0.4±0.02 | 0.3±0.02 | 0.3±0.01 |
| *α*-thujene **^†^** | ― | ― | ― | ― | ― | ― | 50.7±1.9 | 42.9±0.5 | 42.8±2.8 |
| copaene **^†^** | ― | ― | ― | ― | ― | ― | 0.2±0.02 | 0.6±0.1 | 0.4±0.1 |
| longifolene **^†^** | ― | ― | ― | ― | ― | ― | 1.2±0.1 | 0.2±0.01 | 0.3±0.1 |
| sabinene **^†^** | ― | ― | ― | ― | ― | ― | 5.3±0.3 | 4.1±0.2 | 4.5±0.3 |
| *β*-cubebene **^†^** | ― | ― | ― | ― | ― | ― | 0.4±0.1 | 0.4±0.05 | 0.5±0.1 |
| *β*-selinene **^†^** | ― | ― | ― | ― | 4.3±2.0 | 2.0±0.1 | ― | ― | ― |
| *α*-selinene **^†^** | ― | ― | ― | ― | 0.8±0.1 | ― | 0.6±0.1 | 1.2±0.1 | ― |
| *γ*-terpinene | ― | ― | ― | ― | 0.4±0.04 | 0.7±0.2 | 0.5±0.03 | 0.3±0.01 | 0.5±0.04 |
| *α*-muurolene **^†^** | ― | ― | ― | ― | ― | ― | 0.1±0.01 | 0.2±0.01 | 0.1±0.04 |
| *α*-humulene **^†^** | ― | ― | ― | ― | ― | ― | 4.7±0.5 | 7.2±0.6 | 4.8±1.1 |
| *γ*-cadinene **^†^** | ― | ― | ― | ― | ― | ― | 0.4±0.1 | 0.5±0.04 | 0.7±0.3 |
| cadinene **^†^** | ― | ― | ― | ― | ― | ― | 1.1±0.1 | 0.9±0.1 | 1.2±0.3 |
| germacrene D **^†^** | ― | ― | ― | ― | ― | ― | 3.4±0.4 | 4.1±0.5 | 2.8±0.5 |
| *α*-phellandrene | ― | ― | ― | ― | ― | ― | 2.4±0.1 | 1.7±0.1 | 2.3±0.1 |
| limonene | ― | ― | ― | 91.8±0.7 | 83.5±2.6 | 87.2±0.7 | 1.1±0.1 | 1.0±0.1 | 1.3±0.1 |
| (*E*)-*β*-ocimene | 71.1±5.1 | 8.0±2.5 | 65.4±5.3 | ― | 0.4±0.3 | 1.1±0.2 | ― | 0.1±0.01 | 4.7±2.4 |
| (*Z*)-*β*-ocimene | ― | ― | ― | 4.4±0.5 | 2.4±0.3 | 2.9±0.5 | ― | ― | ― |
| (*E,Z*)-2,6-dimethyl-2,4,6-octatriene**^†^** | ― | ― | ― | 3.8±0.2 | 2.1±0.2 | 2.8±0.4 | ― | ― | ― |
| *β*-caryophyllene | ― | ― | ― | ― | 2.0±0.7 | 1.1±0.2 | 1.9±0.1 | 2.5±0.2 | 2.8±0.6 |
| DMNT ^§^ | 28.9±5.1 | 2.1±0.7 | 11.8±2.9 | ― | ― | ― | ― | ― | 1.4±0.8 |
| linalool | ― | ― | 1.3±0.3 | ― | ― | ― | 0.3±0.1 | 0.3±0.02 | 0.1±0.02 |
| (*E*,*E*)-*α*-farnesene | ― | ― | 3.0±0.3 | ― | ― | ― | ― | ― | 0.3±0.1 |
| **Oximes** |  |  |  |  |  |  |  |  |  |
| syn -3-methylbutanal oxime | ― | ― | 2.7±0.2 | ― | ― | ― | ― | ― | ― |
| anti -3-methylbutanal oxime | ― | ― | 1.7±0.2 | ― | ― | ― | ― | ― | ― |
| **Other compounds** |  |  |  |  |  |  |  |  |  |
| eucalyptol **^†^** | ― | ― | ― | ― | ― | ― | 0.7±0.1 | 0.4±0.03 | 0.7±0.1 |
| UN **^†^**^¶^ | ― | ― | ― | ― | 1.3±0.2 | 0.6±0.1 | ― | ― | ― |
| **Total number of chemicals** | **2** | **4** | **8** | **3** | **11** | **9** | **22** | **23** | **24** |

***** Volatiles present at 0.1% or higher proportions in the headspace samples are listed in the table.

^†^ Compounds were tentatively identified by comparison of their MS-spectra with those of in the NIST02 library (Scientific Instrument Services, Inc., USA).

^‡^ Compounds marked with “―” means under detectable level.

^§^ DMNT: (3*E*)-4,8-dimethyl-1,3,7–nonatriene

^¶^ UN compounds with Molecular Weight 150.
