# Supplementary material for: Plants Attract Parasitic Wasps to Defend Themselves against Insect Pests by Releasing Hexenol
Source: PLoS One. 2007 Sep 5;2(9):e852. doi: 10.1371/journal.pone.0000852 (PMC1955833; doi:10.1371/journal.pone.0000852)
Supplement: Table S4 — Relative amount of volatiles released from Rosaceae and Vitaceae plants by undamaged leaf (UL), mechanically damaged leaf with a blade (MDL), and JA-treated leaf (0.12 MB DOC) [file pone.0000852.s004.doc]

| **Table S4** Relative amount of volatiles released from Rosaceae and Vitaceae plants by undamaged leaf (UL), mechanically damaged leaf with a blade (MDL), and JA-treated leaf | | | | | | | |  |
| --- | --- | --- | --- | --- | --- | --- | --- | --- |
| **Chemical compound*** | **Rosaceae Vitaceae** | | | | | | |  |
|  | ***R. chinensis P. tricuspidata*** | | | | | | |  |
|  | Relative % of compounds in different treatments (means ± SE) | | | | | | |  |
|  | UL | MDL | JA-treated | UL | | MDL | JA-treated | |
| **Green leaf volatiles** |  |  |  |  |  | |  |  |
| hexyl acetate **^†^** | ― | 0.9±0.4 | ― | ― | 4.8±0.7 | | ― |  |
| butanoic acid, (*E*)-2-hexenyl ester **^†^** | ― | ― | ― | ― | 0.2±0.1 | | ― |  |
| (*E*)-2-hexenyl acetate **^†^** | ― | 0.7±0.2 | ― | ― | 2.6±0.6 | | ― |  |
| (*Z*)-3-hexenyl acetate | ― | 22.6±2.1 | 2.0±0.8 | ― | 14.6±3.0 | | ― |  |
| (*Z*)-3-hexenol | ― | 4.5±0.9 | ― | ― | 2.5±1.2 | | ― |  |
| hexenal | ― | ― | ― | 14.6±2.3 | 1.3±0.3 | | ― |  |
| (*E*)-2-hexenal | ― | 1.0±0.4 | 1.6±1.7 | ― | 10.8±2.4 | | ― |  |
| (*Z*)-3-hexenal **^†^** | ― | 0.9±0.1 | 0.4±0.2 | ― | 3.2±1.8 | | ― |  |
| (*E*)-2-hexenol | ― | 1.4±0.5 | ― | ― | 0.9±0.2 | | ― |  |
| 1-hexenol **^†^** | ― | 1.2±0.4 | ― | ― | 1.4±0.3 | | ― |  |
| **Terpenoid** |  |  |  |  |  | |  |  |
| *β*-myrcene | ― | 0.4±0.1 | 0.5±0.2 | ― | ― | | ― |  |
| *β*-pinene | 27.7±1.6 | 3.5±0.9 | 2.9±1.1 | ― | ― | | ― |  |
| *α*-pinene | 41.3±3.0 | 5.4±1.3 | 3.6±1.1 | ― | ― | | ― |  |
| *α*-bergamotene **^†^** | ― | ― | 0.5±0.2 | ― | ― | | ― |  |
| (*E,Z*)-2,6-dimethyl-2,4,6-octatriene**^†^** | ― | ― | ― | ― | 0.3±0.1 | | 0.2±0.1 |  |
| (*Z*)-*β*-ocimene | 2.5±0.6 | 0.7±0.1 | ― | ― | 0.7±0.1 | | 1.0±0.02 |  |
| (*E*)-*β*-ocimene | 14.4±2.5 | 19.3±2.3 | 22.7±1.6 | 79.3±2.8 | 47.4±7.2 | | 68.6±1.3 |  |
| *β*-cubebene **^†^** | ― | ― | ― | ― | 0.3±0.1 | | 0.6±0.1 |  |
| *β*-elemene **^†^** | ― | 0.7±0.3 | 0.6±0.3 | ― | ― | | 1.0±0.4 |  |
| δ-elemene **^†^** | ― | ― | ― | ― | ― | | 0.6±0.2 |  |
| limonene | 10.1±2.6 | 1.4±0.5 | 3.0±1.6 | ― | ― | | ― |  |
| *β*-caryophyllene | 4.0±1.7 | 1.0±0.6 | 0.7±0.6 | ― | ― | | ― |  |
| (*E*,*Z*)-4,8,12-trimethyl-1,3,7,11-tridecatetraene**^†^** | ― | ― | ― | ― | ― | | ― |  |
| DMNT ^§^ | ― | 21.6±4.6 | 4.3±1.0 | ― | 3.9±0.9 | | 6.2±0.6 |  |
| TMTT ^¶^ | ― | ― | 0.2±0.1 | ― | ― | | ― |  |
| linalool | ― | 0.5±0.2 | ― | ― | 0.5±0.2 | | ― |  |
| (*E*,*E*)-*α*-farnesene | ― | 7.6±1.8 | 28.7±4.5 | 6.1±1.6 | 3.9±1.5 | | 8.0±0.9 |  |
| **Oximes** |  |  |  |  |  | |  |  |
| syn -2-methylbutanal oxime | ― | ― | ― | ― | ― | | 3.1±0.4 |  |
| anti -2-methylbutanal oxime | ― | ― | ― | ― | ― | | 0.9±0.1 |  |
| syn-3-methylbutanal oxime | ― | ― | ― | ― | 0.2±0.1 | | 2.9±0.3 |  |
| anti-3-methylbutanal oxime | ― | ― | ― | ― | 0.1±0.1 | | 2.0±0.2 |  |
| **Other compounds** |  |  |  |  |  | |  |  |
| methyl salicylate | ― | 0.5±0.2 | 0.3±0.1 | ― | ― | | ― |  |
| 3-methylbutanenitrile **^†^** | ― | ― | ― | ― | ― | | 1.1±0.1 |  |
| 2-methylbutanenitrile **^†^** | ― | ― | ― | ― | ― | | 0.2±0.1 |  |
| 1-nitropentane **^†^** | ― | ― | ― | ― | ― | | 0.5±0.04 |  |
| dodecane | ― | ― | ― | ― | 0.2±0.1 | | ― |  |
| 3-methylbutyl acetate **^†^** | ―^‡^ | ― | ― | ― | ― | | 1.0±0.2 |  |
| 2-methylbutanoic acid,ethyl ester **^†^** | ― | ― | 8.1±1.9 | ― | ― | | ― |  |
| butanoic acid, ethyl ester **^†^** | ― | 1.6±0.8 | 10.9±2.8 | ― | ― | | ― |  |
| hexanoic acid, ethyl ester **^†^** | ― | ― | 6.5±0.8 | ― | ― | | ― |  |
| benzoic acid, ethyl ester **^†^** | ― | 2.6±0.8 | 1.6±0.7 | ― | ― | | ― |  |
| 3-hexenoic acid, ethyl ester **^†^** | ― | ― | 0.7±0.3 | ― | ― | | ― |  |
| 2-hexenoic acid, methyl ester **^†^** | ― | ― | ― | ― | 0.3±0.1 | | ― |  |
| 2-methylbutanol **^†^** | ― | ― | ― | ― | ― | | 0.7±0.4 |  |
| 3-methylbutanol **^†^** | ― | ― | ― | ― | ― | | 0.5±0.2 |  |
| eucalyptol **^†^** | ― | ― | ― | ― | ― | | 0.8±0.4 |  |
| **Total number of chemicals** | **6** | **22** | **20** | **3** | **21** | | **19** |  |

***** Volatiles present at 0.1% or higher proportions in the headspace samples are listed in the table.

^†^ Compounds were tentatively identified by comparison of their MS-spectra with those of in the NIST02 library (Scientific Instrument Services, Inc., USA).

^‡^ Compounds marked with “―” means under detectable level.

^§^ DMNT: (3*E*)-4,8-dimethyl-1,3,7–nonatriene.

^¶^ TMTT: (3*E*,7*E*)-4,8,12-trimethyl-1,3,7,11-tridecatetraene.
