# Supplementary material for: Plants Attract Parasitic Wasps to Defend Themselves against Insect Pests by Releasing Hexenol
Source: PLoS One. 2007 Sep 5;2(9):e852. doi: 10.1371/journal.pone.0000852 (PMC1955833; doi:10.1371/journal.pone.0000852)
Supplement: Table S5 — Plants used in the headspace experiments. (0.04 MB DOC) [file pone.0000852.s005.doc]

Table S5 Plants used in the headspace experiments

| Family | Genus | Species | Common name | Cultivar | From |
| --- | --- | --- | --- | --- | --- |
| Fabaceae | *Phaseolus* | *vulgaris* | kidney bean | Naibai | a |
|  | *Phaseolus* | *lunatus* | Lima bean | L. cv. Ferry Morse var. Jackson Wonder Bush | b |
|  | *Vigna* | *unguiculata* | cowpea | Fengjiang No. 1 | c |
| Solanaceae | *Capsicum* | *annuum* | bell pepper | 78-9 | c |
|  | *Solanum* | *lycopersicum* | tomato | Yuanyi L-402 | c |
| Cucurbitaceae | *Cucumis* | *sativus* | cucumber | Zhongnong No. 16 | c |
| Asteraceae | *Calendula* | *officinalis* | marigold | Xiaolun | d |
| Apiaceae | *Apium* | *graveolens* | celery | Wentula | c |
| Rosaceae | *Rosa* | *chinensis* | Chinese rose | Mingxing | d |
| Vitaceae | *Parthenocissus* | *tricuspidata* | Boston ivy | Unknown | e |

^a^ Haizhong Vegetables Market, Beijing, China; ^b^ provided by Dr. B. Schulze Max Planck Institute for Chemical Ecology, laboratory of Prof. W. Boland, Jena, Germany；^c^ Institute of Vegetables and Flowers, Chinese Academy of Agricultural Science, Beijing, China；^d^ donated by Dr. Jing of Institute of Botany, Chinese Academy of Science, Beijing, China; ^e^ collected locally in the garden of Institute of Zoology, Chinese Academy of Science, Beijing, China.
