# Supplementary material for: Plants Attract Parasitic Wasps to Defend Themselves against Insect Pests by Releasing Hexenol
Source: PLoS One. 2007 Sep 5;2(9):e852. doi: 10.1371/journal.pone.0000852 (PMC1955833; doi:10.1371/journal.pone.0000852)
Supplement: Figure S1 — Absolute amounts of 6 principal induced volatile compounds from 1A, headspace collections of mechanically damaged plants, and from 1B, leafminer-damaged host plants or JA-treated non-host plants. The amounts of volatile released from JA-treated plants were expressed as nanogram per 10 gram fresh weight (FW) per 1h. (1.20 MB DOC) [file pone.0000852.s006.doc]

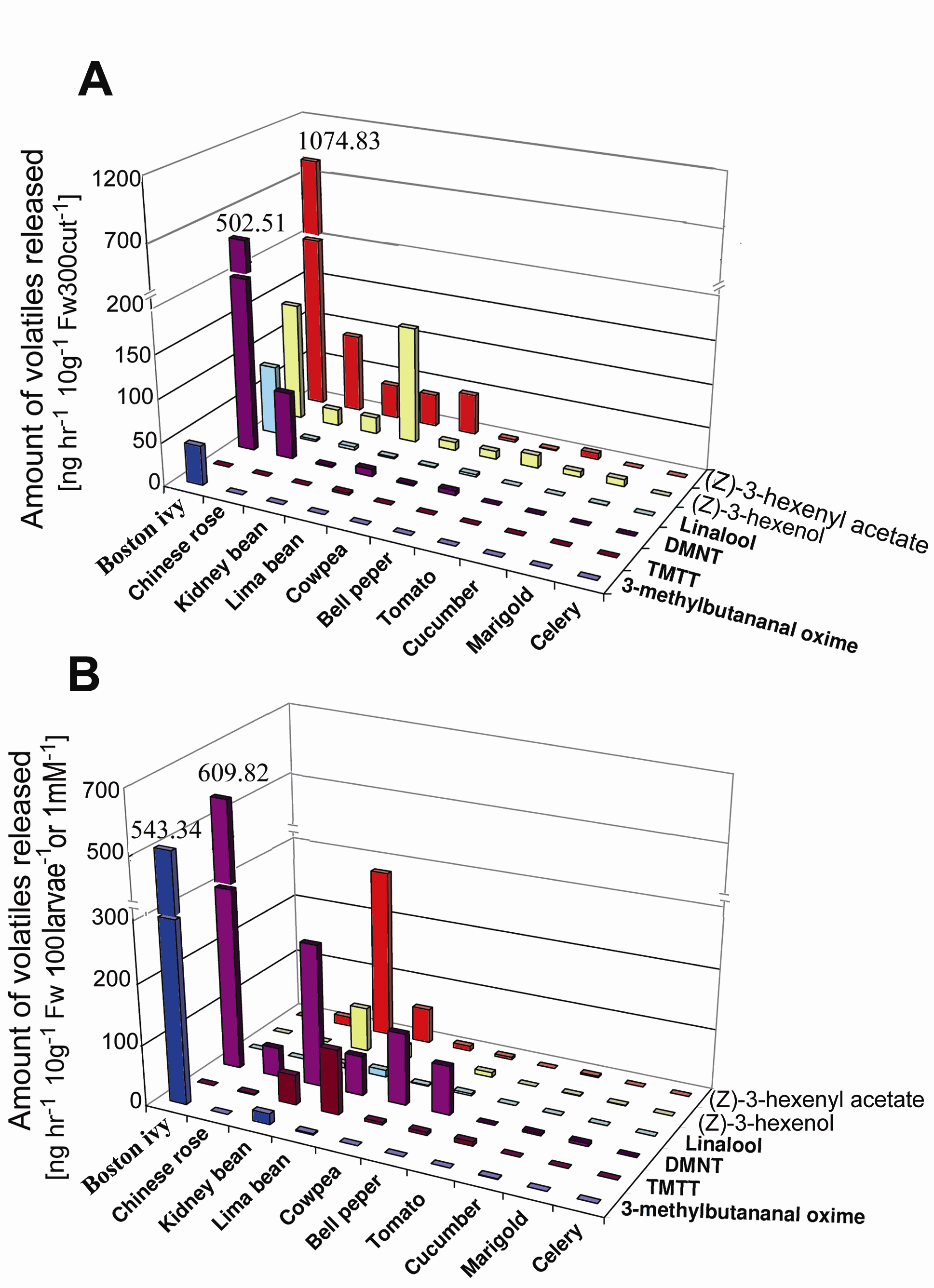


**Figure S1** Absolute amounts of 6 principal induced volatile compounds from **1A**, headspace collections of mechanically damaged plants, and from **1B**, leafminer-damaged host plants or JA-treated non-host plants. The amounts of volatile released from JA-treated plants were expressed as nanogram per 10 gram fresh weight (FW) per 1h.
